# Compulsive Sexual Behaviour Disorder, Aggression, Antisocial Personality, and Spirituality in Correctional and Residential Substance Treatment Populations: A Comparative Correlational Study

**DOI:** 10.1192/j.eurpsy.2026.10454

**Published:** 2026-07-31

**Authors:** E. L. Allagoa

**Affiliations:** 1 Department of Psychiatry, Jos University teaching Hospital, Jos, Nigeria; 2 Department of Psychiatry, Lancaster University, Lancaster; 3 Adult CMHT, Lancashire and South Cumbria NHS Foundation, Preston, United Kingdom

## Abstract

**Introduction:**

Compulsive Sexual Behavior Disorder (CSBD), also termed Hypersexual Disorder (HSD), was recently included in ICD-11 but remains under-studied in correctional and substance use disorder (SUD) populations. These groups may face elevated risk for CSBD, aggression, antisocial personality disorder (ASPD), recidivism, and relapse. The potential protective role of spirituality is also unclear.

**Objectives:**

To compare CSBD/HSD, aggression, ASPD, and spirituality between incarcerated persons and residents in SUD treatment centres; to assess associations between CSBD, access to sexual activity, length of stay, and relapse/recidivism; and to examine relationships between aggression, ASPD, and spirituality.

**Methods:**

A random sample of 208 individuals—matched by age and gender—from a medium-secure correctional facility and two SUD rehabilitation centres (in sobriety, without comorbid mental or medical disorders) in jOs, Northern Nigeria participated. Measures included the Compulsive Sexual Behavior Inventory, Hypersexual Disorder Inventory, Modified Overt Aggression Scale, MINI-Plus modules J (ASPD) and K (SUD), and the Spiritual Involvement and Belief Scale. Comparative analyses examined group differences. Chi-square tested associations with CSBD and ASPD, logistic regressions identified predictors, and Spearman’s correlation assessed strength and direction of associations.

**Results:**

Prevalence rates: CSBD 38.9%, HSD 28.1%, ASPD 42%. All were significantly higher among incarcerated participants. In this group, both length of stay and access to sexual activities predicted CSBD; in SUD treatment, only length of stay was predictive. Across the total sample, relapse/recidivism correlated negatively with CSBD (r = –0.516). No significant differences emerged for spirituality or aggression between groups. Aggression correlated weakly with ASPD (r = 0.114). Spirituality correlated strongly negatively with ASPD (r = –0.990) and CSBD (r = –0.94).

**Conclusions:**

Incarcerated persons show higher CSBD/HSD and ASPD rates than those in SUD treatment. Predictors of CSBD differ by setting. Spirituality appears protective against both CSBD and ASPD. Recommendations include raising awareness among clinicians, health education for high-risk groups, implementing multidisciplinary, evidence-based interventions, and policy reforms to strengthen targeted mental health programs in prisons and rehabilitation centres. Longitudinal studies are needed to clarify causality across the lifespan.

**Keywords:**

CSBD; HSD; SUD; ASPD; Aggression; Spirituality; Incarceration; Rehabilitation

**Disclosure of Interest:**

None Declared

